# The Role of a Low Fermentable Oligosaccharides, Disaccharides, Monosaccharides, and Polyol Diet in Nonceliac Gluten Sensitivity

**DOI:** 10.1155/2018/1561476

**Published:** 2018-08-06

**Authors:** P. Priyanka, S. Gayam, J. T. Kupec

**Affiliations:** ^1^Department of Medicine, West Virginia University Hospitals, Morgantown, WV, USA; ^2^Department of Medicine, Section of Digestive Diseases, West Virginia University Hospitals, Morgantown, WV, USA

## Abstract

**Background:**

Nonceliac gluten sensitivity (NCGS) is a recently defined clinical entity characterized by intestinal and extraintestinal symptoms associated with gluten ingestion in individuals in whom celiac disease (CD) or wheat allergy (WA) has been excluded. Despite its name and definition, gluten has been shown to precipitate symptoms in only 16–30% of these patients. In addition to gluten, other components of wheat, including fermentable oligosaccharides, disaccharides, monosaccharides, and polyols (FODMAPs), alpha-amylase trypsin inhibitors (ATIs) and wheat germ agglutinin have been implicated in the causation of the symptoms of NCGS, with FODMAPs garnering the most attention. We present a review of the existing literature evaluating the role of FODMAPs in NCGS symptomatology.

**Methods:**

A systematic review of PubMed, Cochrane, EMBASE, and Google Scholar for keywords fructans, non-celiac gluten sensitivity, NCGS, FODMAPs, and gluten-free diet (GFD) was conducted through a series of advanced searches. Articles related to the use of fructans or FODMAPs were analyzed.

**Results:**

FODMAPs were found to be associated with gastrointestinal and extraintestinal symptoms in NCGS.

**Conclusions:**

A low FODMAP diet has potential for improvement of clinical symptoms in NCGS. In addition, some evidence suggests an additional benefit to simultaneous adherence to both low FODMAP diet and GFD.

## 1. Introduction

Gluten avoidance has become a popular health trend with nearly 30% of adults avoiding gluten or limiting their intake. Despite conflicting evidence regarding the existence of nonceliac gluten sensitivity (NCGS) as an entity amongst clinicians, it has found prompt and easy acceptance in the general public [1]. NCGS is defined by the presence of intestinal and extraintestinal symptoms related to ingestion of gluten-containing foods in subjects not affected by either celiac disease (CD) or wheat allergy (WA) [2]. Indirect evidence suggests that NCGS could be more prevalent than celiac disease [3]. As per Salerno Experts' Criteria established in 2015, “in the absence of sensitive and specific biomarkers, a closed and standardized monitoring of the patient during elimination and reintroduction of gluten is the most specific diagnostic approach and hence could be used as diagnostic hallmark of NCGS” [2]. There is a significant overlap between the gastrointestinal symptoms of NCGS and irritable bowel syndrome (IBS). The extraintestinal manifestations of NCGS (lack of well-being, fatigue, headache, brain fog, anemia, anxiety, and numbness) respond to dietary modifications and differentiate it from IBS [2]. Additionally, it is recommended that gluten-unresponsive patients should be investigated for other etiologies of IBS-like symptoms including fermentable oligosaccharides, disaccharides, monosaccharides, and polyols (FODMAP) that include fructose, lactose, fructans, galactans, xylitol, sorbitol, maltitol, and mannitol intolerance and small intestinal bacterial overgrowth (SIBO). Recent studies suggest that despite following a long-term gluten-free diet (GFD) in NCGS, milder clinical symptoms may still persist [4].

Although the etiology of NCGS remains unknown, the role of FODMAPs is being increasingly investigated. FODMAPs have been postulated to precipitate functional gastrointestinal (GI) symptoms by inducing distention of GI lumen through their osmotic effects and production of gas in the small bowel and proximal colon related to rapid fermentation by gut bacteria in subjects with visceral hypersensitivity or GI motility disorders [5, 6]. Over the last few decades, due to the increasing westernization of food habits, diet patterns have changed to include FODMAPs in significant amounts. A diet low in FODMAPs has been shown to improve symptoms in patients with IBS, with 70% of patients who follow a low FODMAP diet experiencing significant improvement in symptoms, particularly abdominal pain and distention [7]. Recommendations for a low FODMAP diet were included in the guidelines of the British Dietetic Association in 2010 and 2011 and in the Australian guidelines for the treatment of IBS [8].

This review includes studies on patients with NCGS in whom FODMAPs either directly precipitated symptoms or adherence to low FODMAP diet improved symptoms. In addition, we sought to determine if NCGS is a heterogeneous entity that consists of patients who may improve on low FODMAP diet with or without following a GFD.

## 2. Methods

Relevant articles were identified by systematically searching the Cochrane Library, EMBASE, and PubMed for English language articles published by April 30, 2018. Manual search for relevant publications from the references of extracted articles was also performed. No publication date or publication status restrictions were applied. Preferred reporting items for systemic reviews and meta-analyses (PRISMA) guidelines were followed to develop a protocol including eligibility criteria, search strategies, criteria for study selection, methods for data extraction, and assessing study quality and statistics [9].

Full text of these citations was retrieved and examined in more detail. Six studies were finally included for this review, as shown in Table 1.

## 3. Results

### 3.1. Study Design and Inclusion/Exclusion Criteria (Table 1)

All included studies were original articles. Only one study was open in design, and the remaining studies were randomized, double-blind controlled trials; four were placebo controlled, and five had a crossover design. The primary inclusion criteria were adult patients with self-reported NCGS. In all, a total of 197 patients across all studies were included. The sample size of the studies included varied from 22 to 59 patients.

### 3.2. Gastrointestinal Symptoms

The abdominal symptoms and bloating were associated with fructan challenge in a recent randomized, double-blind, placebo-controlled, crossover challenge (DBPCC) study involving 59 subjects with self-reported NCGS, as measured by gastrointestinal symptom rating scale-irritable bowel syndrome (GSRS-IBS) and GSRS bloating score. Also, visual analogue scale (VAS) for pain, bloating, flatus, nausea, and stool dissatisfaction was higher in the fructan challenge cohort [10]. In an Italian study, GI symptoms noted in both control and test groups could be attributed to the presence of FODMAPs in both flours [11]. The presence of FODMAPs was substantially higher in the gluten-free flour with 6.8 g lactose, 0.16 g fructans, and 0.04 g sucrose, compared to gluten-containing amygluten with 0.8 g fructans, 0.2 g sucrose, and 0.08 g fructose. It is plausible that the symptoms of the gluten-free diet were caused by the high FODMAP content and thus cannot be distinguished from true clinical symptoms after gluten stimulation. An eight-week low FODMAP diet stage of another study by the same group showed that all five dimensions of the GSRS were reduced [12]. This study concluded that the patients reporting gluten sensitivity are a heterogeneous population composed of true gluten sensitivity and FODMAP sensitivity.

In an Australian DBPCC involving 37 patients, after a two-week run in period of low FODMAP diet and GFD, NCGS patients had significant and consistent improvement in abdominal pain, bloating, and satisfaction with stool consistency, flatus, and fatigue. Similar findings were noted in the rechallenge stage of the trial [13]. In another similar study, NCGS patients were placed on FODMAP restriction followed by GFD [14]. On the low FODMAP diet, GSRS improved significantly for reflux, abdominal pain, and indigestion. These symptoms improved further during the GFD stage of the trial. This study suggested an additional benefit of GFD with low FODMAP diet. Similarly, in another trial, studying the effect of gluten challenge in NCGS patients, there was no observable difference in the GI symptoms of the control and test cohorts, once FODMAPs were removed from both gluten-free and gluten-containing flours [15]. These studies help establish the causative role of FODMAPs in the GI symptoms of NCGS.

### 3.3. Extraintestinal Symptoms

#### 3.3.1. Vitality

Health-related quality of life indicated the lowest score for the “vitality” subdimension during fructan challenge in a Norwegian study [10].

#### 3.3.2. Fatigue

Fatigue and weakness were significantly higher after fructan challenge and not different between gluten and placebo arms [10]. Similarly, fatigue was associated with FODMAP use and improved with the elimination of FODMAPs in other studies [11–13].

#### 3.3.3. Depression

Higher depression scores were noted in subjects challenged with gluten following a low FODMAP diet and GFD [15]. Psychological parameters improved remarkably initially after the initiation of a low FODMAP diet and further improved on a GFD in this study [15].

### 3.4. Nocebo or Negative Placebo Effect of Gluten

A strong nocebo response was seen in all the included studies [16, 17]. In the Norwegian study, symptom response to placebo (*n* = 22) was almost as high as response to fructan challenge (*n* = 24) and significantly higher than the gluten challenge (*n* = 13) cohort [10].

About one-fifth of participants in an Italian study did not report worsening of symptoms after a challenge with either gluten-rich or gluten-free flour [11]. As there was no placebo arm in this trial, the authors speculated that in real-life situations, patients might be experiencing their symptoms due to psychological anticipation of intolerance when exposed to gluten and thereby suggesting a nocebo effect. Although it has been shown that NCGS patients did not show an increased tendency for general somatization, emotional factors may still play a role [18]. Only 8% of participants specifically showed true sensitivity to gluten in trial conditions, much lower than real-life conditions, possibly attributable to the nocebo effect [13].

The strong nocebo effect raises the concern of feasibility or even the usefulness of a DBPCC in clinical practice for the diagnosis of NCGS.

### 3.5. Biomarkers

In an emerging area of study, the use of biomarkers in monitoring response in NCGS is uncommon [13, 14]. In one study, only one participant was found to have a positive T-cell response after a high-gluten challenge. No significant difference across the treatment groups for other biomarkers including eosinophil cationic protein, radio allegro-sorbent test, fecal pH and fecal concentrations of human *β*-defensin-2, calprotectin, and ammonia levels was noted [13]. In another study, intraepithelial lymphocytes (IELs) were moderately increased in roughly 42% patients with NCGS, but the extent was lower than typically seen in CD [14]. IELs and goblet cells reduced significantly on GFD as compared to baseline, thereby suggesting the added benefit of GFD.

### 3.6. Intestinal Microbiota

Only one trial examined changes in the gut microbiota [14]. In healthy controls, relative to NCGS patients, colonies of bacteria belonging to the phylum Bacteroidetes were higher, and phylum Firmicutes were lower compared to NCGS patients. Similar findings with phylum dysbalance have been observed in IBS patients [19, 20]. In NCGS patients, a significant increase in Bacteroidetes and a reduction of Firmicutes were noted with a GFD compared to a low FODMAP diet. This study highlighted that the microbiota from NCGS patients are more susceptible to the various dietary modifications compared to healthy controls. GFD also was associated with a significant increase in Bacteroidetes compared to low FODMAP diet. Notably, while healthy controls did not display any significant variations in microbiotic signatures, NCGS patients displayed significant changes in bacteria responsible for dehalogenation, ammonia oxidation, xylan and cellulose degradation, sulfate reduction, and nitrogen fixation especially while adhering to GFD. These effects were less prominent with low FODMAP diet in this study. These findings strengthen the evidence for the additive benefits of low FODMAP diet and GFD.

### 3.7. Discussion

Our review presents the evidence that NCGS patients could potentially benefit from FODMAP restriction with or without gluten restriction. The results suggest that a subset of NCGS patients actually has FODMAP intolerance. This raises the question of NCGS as an entity specifically used in the context of gluten sensitivity as well as its distinction from FODMAP intolerance. FODMAPs may be a causative factor in GI symptoms and to some extent in extraintestinal symptoms such as fatigue and loss of vitality in some NCGS patients. NCGS may be a heterogeneous entity with multiple factors such as FODMAPs in addition to gluten contributing to symptom generation.

In a landmark study that established the current existence of NCGS as a separate entity, gluten was shown to induce both GI and extraintestinal symptoms in patients without CD [21]. Notably, in this study test, gluten was devoid of FODMAPs. In another study from the same group, subjects who were already slightly improved on GFD improved further on a low FODMAP diet [13]. Also, they failed to worsen with a gluten challenge. A strong association of FODMAPs with GI symptoms such as bloating and indigestion was noted across all studies. This correlation was noted in multiple ways like symptomatic improvement on low FODMAP diet or worsening of symptoms with a fructan challenge or the equal presence of symptoms in both placebo and study cohorts if they were both being exposed to fructans [10–15].

Extraintestinal manifestations of NCGS, such as fatigue, depression, and anxiety, were mostly evaluated as secondary outcomes. Fatigue and vitality were significantly worse after a fructan challenge in some studies [10, 12]. Furthermore, fatigue improved significantly with a FODMAP-free run-in period and subsequently increased during the dietary challenge arm, irrespective of the dietary challenge. The extraintestinal symptoms in these patients could possibly be attributed either independently to the neurological effects of gluten present in wheat or combined effect of FODMAPs and gluten as in real-life scenarios. A low FODMAP diet followed by 14 days on GFD resulted in reduced IELs indicating the additive benefit of the two diets. Since intestinal biopsies were not performed immediately after low FODMAP diet in this study, it is hard to comment if the reduction was due to adherence to GFD or low FODMAP diet. These investigators suggested that NCGS patients could benefit from following low FODMAP and GFD simultaneously. The cumulative, beneficial effects of low FODMAP diet and GFD on gut microbiota suggest that adherence to both diets may prove superior to choosing to follow only one diet [14].

Similar synergic effects of a low FODMAP diet and GFD were noted in other studies [10, 11, 13, 15].

Increasing evidence indicates that only a very small percentage (16–30%) of patients were actually found to have NCGS in rechallenge studies. Two separate meta-analyses of double-blind placebo-controlled gluten challenge trials in NCGS explore the possibility of gluten not being responsible for symptoms in self-reported NCGS [1, 22]. In one meta-analysis, the percentage of relapse correlated significantly with the amount of gluten used and the duration of the challenge [22]. The same study also found that if Salerno Criteria were strictly followed, the percentage of relapse was notably higher (up to 40%) after gluten challenge when compared to placebo. Another meta-analysis concluded that gluten may not be responsible for the intestinal and extraintestinal symptoms in a large majority of patients with self-reported NCGS [1]. In addition, these authors argued that the Salerno Experts' Criteria (an expert committee recommendation, not evidence-based) may be an imperfect tool to diagnose NCGS. Since a prominent nocebo effect was uniform to all studies, it could be argued that the very design of the studies (a placebo control) could have contributed to the effects noted. Carryover effects in crossover trials or placebos containing unintentional substances that could precipitate symptoms are some alternate explanations of the observed nocebo effect. These authors also proposed a “melting pot hypothesis” for NCGS. In this hypothesis, between the two distinct entities of CD and WA are various entities including the “gluten sensitivity” group, wheat-induced symptom group (with negative results after a gluten challenge, nocebo effect), and FODMAP intolerance group [1]. They also emphasized the importance of accurately excluding CD as it was inadequately ruled out in approximately 61% patients in one survey [23]. These authors recommended more sensitive assays (than the usual testing) be used for patients with gluten-related symptoms and HLA DQ2/8 haplotypes [24, 25]. They also considered FODMAP intolerance as an important subgroup in the grey zone patients between CD and WA. The distinction between patient groups is clinically important as truly gluten-sensitive patients must adhere to a GFD and patients with FODMAP intolerance could benefit from low FODMAP diet, not necessarily gluten restriction.

Despite the success of these diets in study conditions, or even clinically, adherence to a restrictive diet like low FODMAP should always be initiated and monitored by a registered dietician trained in this area. As a concept, low FODMAP diet is complex, and it has always been meant to be a dietician-delivered diet [26]. This would potentially avoid nutritional deficiencies including lower fiber and calcium in the followers of this diet. In addition, this would present as an opportunity to personalize the diet to patients' individual, specific dietary sensitivities. Despite all the negative GI effects seen in susceptible NCGS and IBS patients, FODMAPs have many beneficial effects on the colon including “prebiotic effects” as they selectively stimulate the growth and activity of potentially beneficial colonic bacteria, specifically *Bifidobacteria* and *Lactobacilli* [27, 28]. In addition, FODMAPs are fermented in the gut to short-chain fatty acids by bacteria that have a trophic effect on the colonic epithelium and protective effect against the colon cancer [29, 30]. Dietary FODMAPs increase stool bulk, improve calcium absorption, modulate immune function, and decrease serum cholesterol, triglycerides, and phospholipids [31]. A low FODMAP diet may also be deficient in natural antioxidants like flavanoids, carotenoids, and vitamin C contained in vegetables like cauliflower, onion, and garlic and phenolic acid and anthrocyanins in fruits like blackberries [31]. At this time, it is also not clear if NCGS is a transient or a permanent condition. Since the long-term effects of following a low FODMAP diet on other systems and colon carcinogenesis are not known, it must be continued with caution and careful monitoring of adverse effects. Once initiated on this diet, patients should be periodically rechallenged in a graded fashion to identify specific dietary triggers and limits of tolerance [32]. The original proponents of this diet recommend “all FODMAP-free period” of 6–8 weeks, followed by reintroduction of one FODMAP per week [33].

### 3.8. Limitations

First, due to a high nocebo response, the role of a DBPCC, the current gold standard for the diagnosis of NCGS, may be questionable [10]. Even if DBPCC studies produced results that are statistically significant, they may not have sufficient clinical relevance due to prominent nocebo effect seen across the studies. Since NCGS at this time is a poorly defined condition with highly subjective symptoms, a common clinical approach of eliminating suspected symptom-inducing foods followed by clinician-supervised rechallenge with close symptom monitoring has been advocated. This may prove superior to DBPCC due to its ease of administration and being more informative.

Second, the symptomatic effect of gluten with fructans and other components of wheat may be additive or even synergistic. The fructans in the food matrix may give a different clinical response than the study materials (supplements of pure fructans added to muesli bars derived from chicory roots versus real-world wheat sources) [10]. Other wheat proteins like alpha-amylase trypsin inhibitors, lectins, and wheat germ agglutinin may play a role in the causation of the symptoms. Opiate-like effects of gluten and IgE WA are other proposed hypotheses [34, 35]. Current understanding of the pathogenesis of NCGS is quite limited and data is scarce. Third, variable methodological designs make the data heterogeneous and difficult to compare. Fourth, the period of following GFD before entering the study was variable from six weeks to six months. A large placebo effect could be seen with shorter periods of pretrial GFD adherence [11]. Finally, recall bias is common to all the studies that monitor response during gluten challenge, and the studies considered in this review were no exception.

## 4. Conclusions and Future Directions

This review suggests a multifactorial etiology of NCGS. FODMAPs may be responsible for gastrointestinal and extraintestinal symptoms in a subset of patients with NCGS [13]. In addition, some evidence suggests that gluten and FODMAPs together may have additive effects on the clinical symptomatology of NCGS and at least some patients may improve by adhering to low FODMAP diet alone or in combination with GFD. Also, this review highlights the immense potential for specific dietary interventions in NCGS and other related functional GI disorders. Even as our knowledge and understanding of NCGS are still in infancy, the combination of translational studies on potential mechanisms, along with larger, high-quality clinical studies on the role of dietary interventions and possibly revision of criteria to better define NCGS in clinical settings, would help us better understand this elusive and complex condition. As proposed in one of the meta-analysis of NCGS rechallenge studies, “Nonceliac Wheat Sensitivity” might be a more accurate term to cover the spectrum of patients showing sensitivity to wheat through a non-IgE mechanism. Finally, as patients look for answers, our limited knowledge and understanding of NCGS should be honestly discussed with them.

## Figures and Tables

**Table 1:** Studies for FODMAPs role in NCGS.

Authors, year, country	Design/method of studying FODMAP effect	Number of subjects (*n*)	CD exclusion method	Protocol	Primary outcome/results	Secondary outcomes/results
Skodje et al., 2018, Norway [10]	RDBPCC fructan challenge (2.1 g), gluten (5.7 g), and placebo given as a muesli bar	*n* = 59 self-reported NCGS	Negative HLA DQ2/DQ8 or normal duodenal biopsy (marsh 0) on GFD if positive for above haplotypes	GFD for 6 m, 7 d on first diet challenge, 7 d washout, then crossover to next arm	GSRS-IBS, recorded for pain, bloating, constipation, diarrhea, satiety Overall GSRS-IBS borderline significant for fructan (38.6) versus gluten (33.1) and placebo (34.3) Significant difference in GSRS-IBS for bloating after fructan	Daily GI symptoms by VAS for overall GI symptoms higher with fructan Health-related quality of life by SF-36 lowest for fructan for vitality Depression and anxiety by Hospital Anxiety and Depression Scale and fatigue by VAS and GSCL highest after fructan

Dieterich et al., 2018, Germany [14]	Open low FODMAP diet adherence for 2 wk	*n* = 19 self-reported NCGS, *n* = 10 healthy controls	IgA/G to TTG and deamidated gliadin peptides, EGD, and duodenal biopsy in NCGS patients	GCD with 10 g gluten for 4 wk, 2 wk low FODMAP diet, then 5 d transition, GFD 2 wk follow up EGD in 17 patients (with persisting symptoms)	Improvement of GI symptoms by GSRS on low FODMAP diet for NCGS pts. for reflux, abdominal pain, and indigestion Further improvement on GFD for abdominal pain, diarrhea, and constipation	Psychological well-being by PGWB improved on low FODMAP diet and further on GFD Reduced IELs on GFD Stool microbiota showed differences in NCGS and controls with reduced *Bifidobacteriae* on low FODMAP diet. Near normalization of gut flora on GFD

Zanini et al., 2015, Italy [11]	RDBPCC GCF had fructans 0.8 g/100 g. GFF had 0.16 g/100 g fructans present in both study arm materials.	*n* = 35 self-reported NCGS	Negative t-TG and/or endomysial antibodies and normal villous structure on duodenal biopsies (marshes 0, 1, 2)	GCF or GFF for 10 days, then 2 wk washout period, then crossed over to another group	GFD for 6 m, ability to identify gluten-containing flour *n* = 12 (34%) Inaccurate, *n* = 17 (49%) Unable to distinguish (17%)	GSRS score for pain, reflux, indigestion, diarrhea, and constipation and VAFS for fatigue increased with GCF in NCGS and GFF in GFF-sensitive. No changes in t-TG IgA and antigliadin IgA and IgG

Zanini et al., 2014, Italy [12]	RDBCC fructans present in both study arm materials and low FODMAP diet for 8 weeks	*n* = 25 self-reported NCGS	Negative t-TG and/or endomysial antibodies and normal villous structure on duodenal biopsies (marshes 0, 1, 2)	GFD, 10 g gluten versus 10 g gluten-free flour for 10 d, then 2 wk washout, then low FODMAP diet for 8 wk	Able to identify gluten-containing flour *n* = 8 Inaccurate, *n* = 12 Unable to distinguish, *n* = 5	GSRS score for pain, reflux, indigestion, diarrhea, and constipation improved on low FODMAP diet with worsening on GFF. VAS for fatigue unchanged with GCF. No changes in t-TG IgA and antigliadin IgA and IgG

Biesiekierski et al., 2013, Australia [13]	RDPBPCC, low FODMAP diet adherence for 2 wk	*n* = 37 IBS patients fulfilling NCGS criteria	Negative HLA DQ2/DQ8 or normal duodenal biopsy (marsh 0) on GFD if positive for the above haplotypes	GFD and 2-week low FODMAP diet, then one of the arms—high gluten (16 g), low gluten (2 g gluten and 14 g whey protein), control for 3 d, washout 2 weeks, crossover 3 d	VAS for overall abdominal symptoms, pain, bloating, wind, stool consistency satisfaction, and tiredness nausea improved in low FODMAP run-in period. 6 (16%) pts. had worsening of overall symptoms in high-gluten arm; only 3 pts. had worsening in placebo arm.	Fatigue with D-FIS was the lowest with low FODMAP diet and worse with all the 3 challenges. No effects on physical activity or sleep by accelerometry in any arm; only 1 subject elicited positive gliadin-specific T-cell response. No significant difference across the arms for ECF protein, RAST, serological markers, fecal wet and dry weight, pH, human *β*-defensin-2, calprotectin, and ammonia levels

Peters et al., 2014, Australia [15]	RDBPCC low FODMAP diet adherence for the entire duration of study	*n* = 22 IBS with improvement on GFD	Negative HLA DQ2/DQ8 or normal duodenal biopsy (marsh 0) on GFD if positive for above haplotypes	GFD and low FODMAP diet for the duration of study followed by 1 of the 3 dietary challenges —gluten, whey, and placebo 3 d, then 3 d crossover to next diet	Depression by STPI worse with gluten versus placebo but similar to whey	GI symptoms by VAS, cortisol levels similar across all the treatment arms

RDBPCC: randomized double-blind placebo-controlled crossover challenge; G: Gram; GSRS-IBS: gastrointestinal symptom rating scale irritable bowel syndrome; VAS: visual analogue scale; VAFS: visual analogue fatigue score; GSRS: gastrointestinal symptom rating scale; GSCL: Giessen Subjective Complaint List; STPI: Spielberger State-Trait Personality Inventory; PGWB: Psychological General Well-Being Index; GCF: gluten-containing flour; GFF: gluten-free flour; GCD: gluten-containing diet; t-TG: tissue transglutaminase; EGD: esophagogastroduodenoscopy; wk: week; IEL: intraepithelial lymphocyte; pt: patient; D-FIS: daily-fatigue impact scale; ECF: eosinophil cationic protein; RAST: radioallergosorbent test; d: days.
